# Tendons from kangaroo rats are exceptionally strong and tough

**DOI:** 10.1038/s41598-019-44671-9

**Published:** 2019-06-03

**Authors:** Mehrdad Javidi, Craig P. McGowan, Nathan R. Schiele, David C. Lin

**Affiliations:** 1 Voiland School of Chemical Engineering and Bioengineering, Washington State University, PO Box 646515, Pullman, WA 99164 USA; 2 Department of Biological Sciences, University of Idaho, 875 Perimeter Drive, MS 3051, Moscow, ID 83844 USA; 3 WWAMI Medical Education Program, University of Idaho, 875 Perimeter Drive, MS 4207, Moscow, ID 83844 USA; 4 Washington Center for Muscle Biology, Washington State University, PO Box 646515, Pullman, WA 99164 USA; 5 Department of Biological Engineering, University of Idaho, 875 Perimeter Dr. MS 0904, Moscow, ID 83844 USA; 6 Department of Integrative Physiology and Neuroscience, Washington State University, PO Box 647620, Pullman, WA 99164 USA

**Keywords:** Bone quality and biomechanics, Biomechanics

## Abstract

Tendons must be able to withstand the forces generated by muscles and not fail. Accordingly, a previous comparative analysis across species has shown that tendon strength (*i*.*e*., failure stress) increases for larger species. In addition, the elastic modulus increases proportionally to the strength, demonstrating that the two properties co-vary. However, some species may need specially adapted tendons to support high performance motor activities, such as sprinting and jumping. Our objective was to determine if the tendons of kangaroo rats (k-rat), small bipedal animals that can jump as high as ten times their hip height, are an exception to the linear relationship between elastic modulus and strength. We measured and compared the material properties of tendons from k-rat ankle extensor muscles to those of similarly sized white rats. The elastic moduli of k-rat and rat tendons were not different, but k-rat tendon failure stresses were much larger than the rat values (nearly 2 times larger), as were toughness (over 2.5 times larger) and ultimate strain (over 1.5 times longer). These results support the hypothesis that the tendons from k-rats are specially adapted for high motor performance, and k-rat tendon could be a novel model for improving tissue engineered tendon replacements.

## Introduction

Tendons are specialized musculoskeletal tissues that experience high mechanical forces generated during motor activities such as running and jumping. To withstand these forces and provide a reasonable safety factor, tendons need to be both strong and tough^1^. Strength and toughness are material properties that describe the maximum amount of stress a material can withstand and the amount of energy it can absorb before it fails or ruptures, respectively. Both properties are important because they in part define the limits of motor function. Namely, operation outside of the limits defined by these properties can cause tendon ruptures and injury resulting in diminished motor performance, leading to mortality for animals living in their natural habitat or diminished quality of life in humans. Given the critical role of tendons in animal survival and human health, we wanted to identify mammalian tendons that have potentially evolved to be stronger and tougher. A tendon that is stronger and tougher than usual could provide a template for developing novel tissue engineered biomaterials for tendon replacements and strategies for improving the functional properties of tendon.

The mechanical properties of tendons associated with a wide range of muscles and species have been well studied^2–4^. Thus, the relative strength and toughness of a specific tendon can be evaluated by a comparative approach. A meta-analysis of 50 studies, with tendons from mammalian species ranging from mice to cows, showed that tendon mechanical properties uniformly vary with body size^5^. In general, increases in body size are associated with an increase in elastic modulus and a proportional increase in ultimate stress (*i*.*e*., strength). This observation has two important implications: (1) the ultimate strain, equal to the ratio of ultimate stress to elastic modulus, is relatively preserved across species, and (2) tendons across all species have similar limitations and constraints when it comes to the covariation of elastic modulus and ultimate stress. Moreover, toughness (the area under the stress-strain curve) also should increase proportionally with elastic modulus, but this relationship was not as well defined and may be impacted by tendon’s non-linear stress-strain relationship. Remarkably, the linear relationship between elastic modulus and ultimate stress is robust (R^2^ = 0.785) across 11 mammalian species despite differences in study methodology, the muscles attached to the tendons, and tendon modifications due to age, injury, genetic alterations, or *in vivo* mechanical interventions (*i*.*e*., disuse, vibration, strength training)^5^. However, some species with highly specialized motor function, such as kangaroo rats, were not included in the meta-analysis, as their tendons have not been studied previously.

Kangaroo rats (k-rats) are small bipedal hoppers (mass approximately 100 g) that can jump vertically ten times their standing hip height^6,7^. To accomplish this extreme level of motor performance, their ankle extensor muscles can generate muscle stresses within the range of those generated by relatively larger animals, such as red kangaroos and wallabies^6,8^. K-rats represent a unique animal that can operate at the extremes of muscle stresses^9^, but the margin by which the tendons involved in jumping, *i*.*e*., the plantaris longus (PL) and gastrocnemius (GAS) tendons, can withstand these forces is unknown^10^. Therefore, the objective of this study was to evaluate the mechanical properties of the PL and GAS tendons of k-rats and directly compare them to the tendons of quadrupedal white rats (referred herein as rat) as a point of reference that is often studied. Based on the jumping performance of k-rats and the implications of the meta-analysis of tendon studies, we expected that k-rat tendons would have an elastic modulus, ultimate stress, and toughness that are large for an animal of its size, and with an ultimate strain comparable to other species. Instead, we found that the elastic moduli of k-rat tendons are not different from rat tendons but have much larger ultimate stress (nearly 2 times larger), much greater toughness (over 2.5 times), and longer ultimate strain (over 1.5 times). These measurements show that k-rats have adapted to independently vary elastic modulus, strength, and toughness in their tendons, which is an exception to the constraints of tendon properties that other animal species follow. This result is significant because it could lead to a new understanding of how tendon properties can be uniquely adapted.

## Results

All results are presented as mean ± standard error of the mean (SEM) of data from ten animals each of k-rats and rats.

### Anatomical measurements

**Figure 1:**
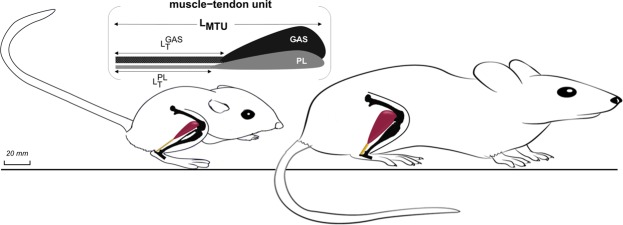
Schematic comparison between the body mass and dimensions of the muscle-tendon units of k-rats (left) and rats (right). Drawing is to scale based upon dimensions given in the text (Table 1). L_MTU_ indicated the combined muscle-tendon lengths, which are equal for k-rats and rats. ${{\rm{L}}}_{{\rm{T}}}^{{\rm{GAS}}}$ and ${{\rm{L}}}_{{\rm{T}}}^{{\rm{PL}}}$ are the tendon length of GAS (combined LG and MG) and PL, respectively.

We compared the size and dimensions of k-rat and rat hindlimb muscle-tendon units for both absolute and normalized values (Fig. 1; dimensions drawn to scale) and found that the k-rats had disproportionally larger muscles and longer tendons. Specifically, the body mass of the rats was three times greater than that of the k-rats (Table 1). The absolute mass of hindlimb muscles (GAS and PL combined) of the rats was also larger than that of the k-rats. However, when normalized by body mass, the muscle mass of the k-rats was significantly larger than muscle mass of the rats (P < 0.0001). There was no significant difference between the absolute muscle-tendon unit length of the muscles (L_MTU_ in Fig. 1; the muscle-tendon unit lengths of the GAS and PL were assumed to be the same due to the similarities of their femoral and calcaneal connections) of the two species (P = 0.29). K-rat PL and GAS tendon lengths normalized to their muscle-tendon unit lengths were significantly longer than those of the rat group (P_PL_ < 0.0001, P_GAS_ < 0.0001).

**Table 1:** Anatomical measurements.

	Body mass (g)	Plantarflexors mass (% body mass)	L_MTU_ (mm)	Tendon length (%L_MTU_)	
				PL	GAS
K-rat	**101 ± 5.0**	**1.36 ± 0.05**	40 ± 1.49	**40 ± 2**	**52 ± 2**
Rat	**318 ± 29.2**	**0.70 ± 0.08**	36.5 ± 1.61	**19 ± 1**	**31 ± 3**

Properties with significant differences between the two species (P < 0.05) are indicated in bold.

### Stress-strain measurements

**Figure 2:**
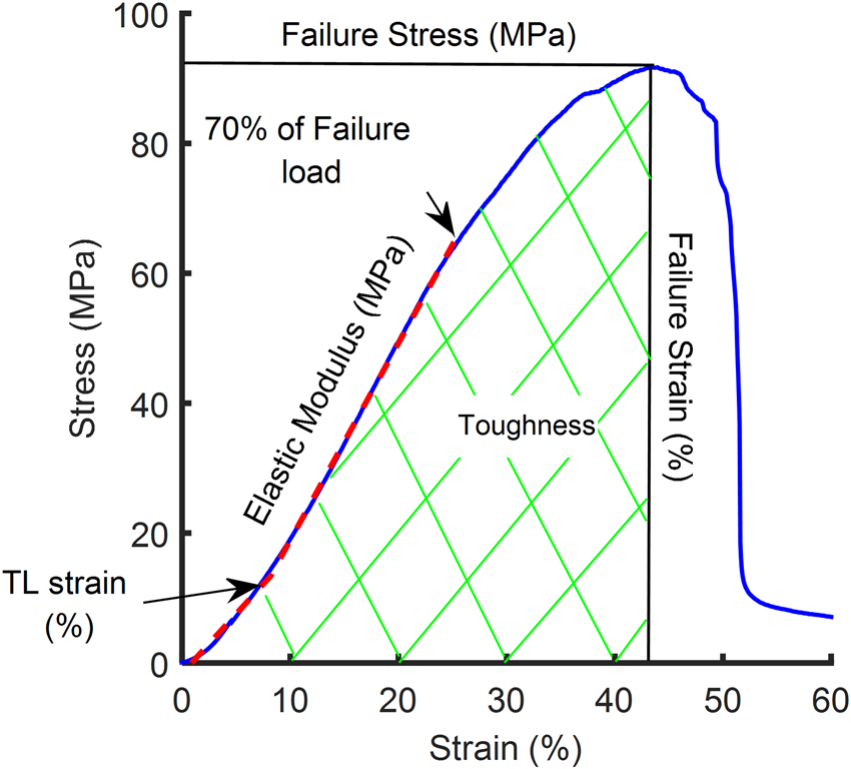
Properties of tendon calculated from stress-strain curves. Bilinear fitted curves (dashed lines) were used for the calculation of toe and elastic stiffness and modulus (R-squares > 0.9 for all 40 tendon samples). TL strain is the toe-to-linear transition strain. Area of the green-hatched region is equal to toughness.

**Figure 3:**
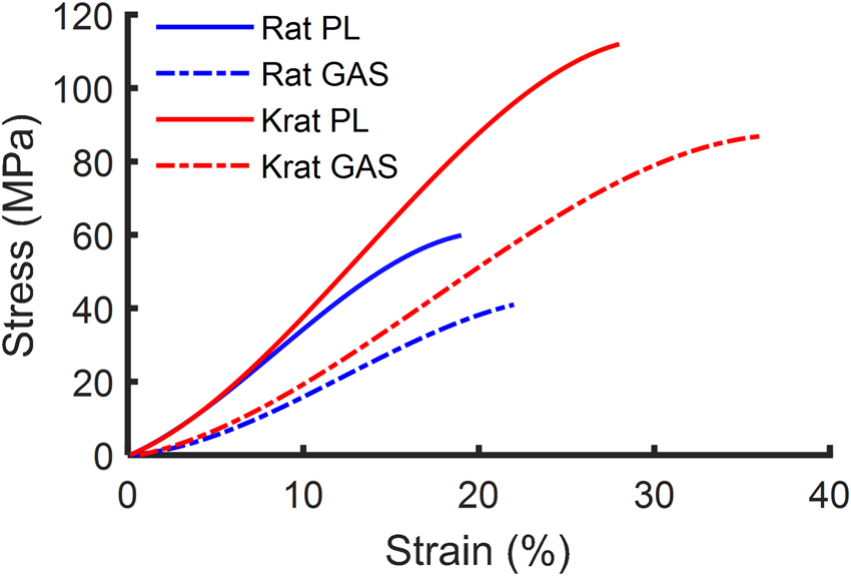
Mean stress-strain curves of each tendon. Mean stress-strain curves of PL (solid) and GAS (dashed) tendons from k-rat (red) and rat (blue) were obtained by fitting 3^rd^ order polynomial curves (R-squares > 0.9) to average stresses over the range of strains indicated in the figure for all 10 samples of each tendon type.

**Figure 4:**
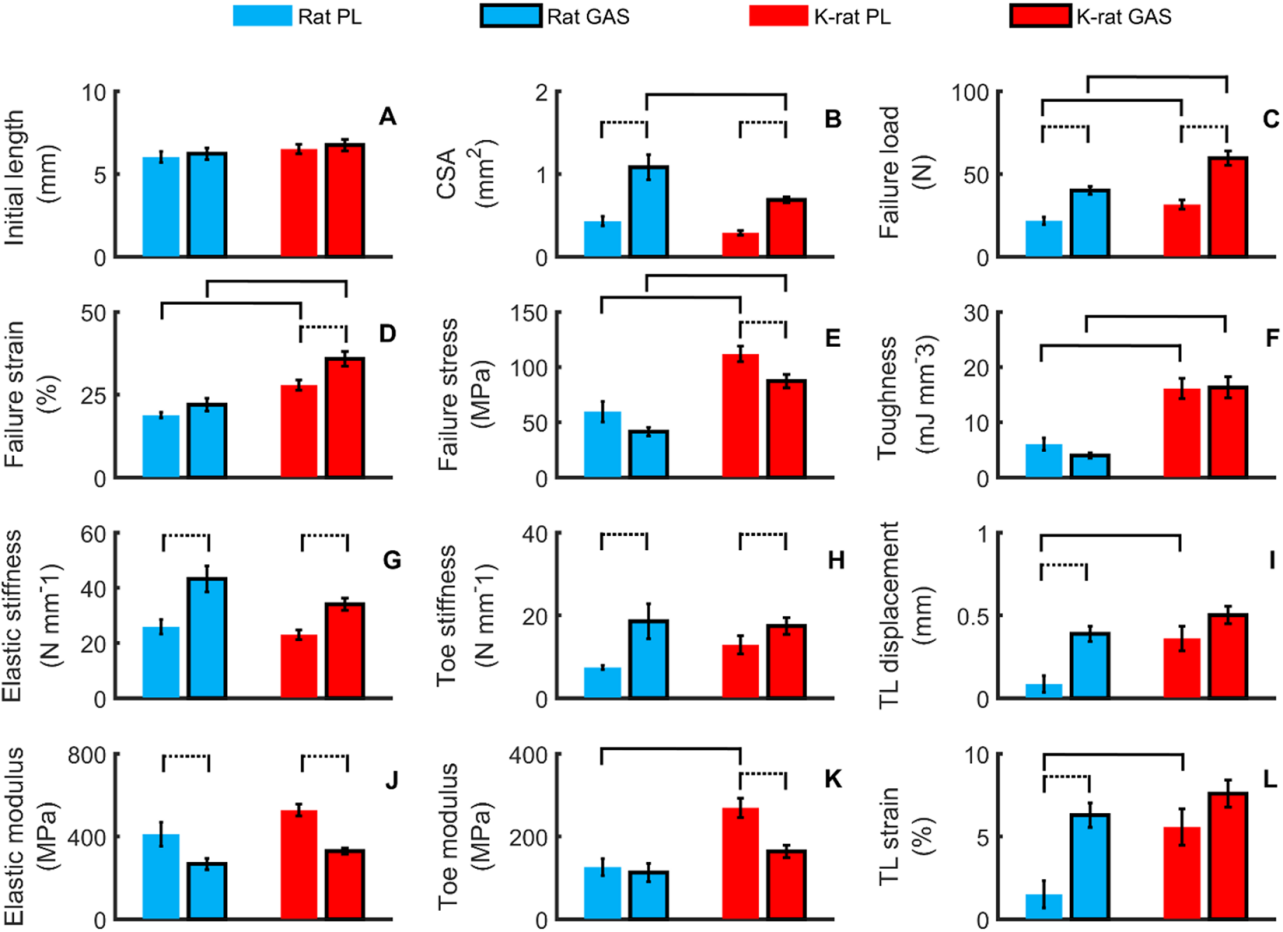
Structural and material properties of rat and k-rat tendons. PL and GAS tendons from rats (blue) and k-rats (red). Shown are mean ± SEM. Data from PL tendons are bars without an outline, and data from GAS tendons are bars with an outline. Solid and dash lines indicate significant differences (P < 0.05) between species and tendon types, respectively.

We measured the stress-strain curve of each tendon by recording the force in the tendon in response to a pull-to-failure applied at a constant strain rate (Fig. 2). The stress was calculated by dividing the force by the tendon’s cross-sectional area (CSA), and strain calculated by dividing the displacement by the tendon’s initial length. The resulting stress-strain curve was well fitted by a bilinear relationship (R-squared > 0.99 for the linear modulus region). We then calculated the mean stress-strain curves of PL and GAS tendons of both species (Fig. 3). All samples for this study were stretched to failure from similar initial length (Fig. 4A). CSA of the rat GAS tendon was larger than that of the k-rat (P = 0.037), while there was no significant difference between the CSA of PL tendons of different species (P = 0.19) (Fig. 4B). The CSA of the GAS tendon of both species was larger than their PL tendon (P_k-rat_ = 0.004, P_rat_ = 0.002).

To statistically test for differences in tendon properties between muscle types and species, we calculated metrics that were both absolute (*i*.*e*., structural properties) and normalized (*i*.*e*., material properties) values (Fig. 4). For between species comparisons, we found that the tendons of the same muscles (GAS or PL) from different species were significantly different in failure values. Specifically, the failure loads of the k-rat PL and GAS tendons were 45% and 49% larger than those of the rat PL and GAS tendons, respectively (P_PL_ = 0.037, P_GAS_ = 0.004) (Fig. 4C). Failure strains of k-rat PL and GAS tendons were 48% and 63% larger than those of rat (P_PL_ = 0.002, P_GAS_ = 0.006) (Fig. 4D). Similar to these structural mechanical properties, failure stress and toughness of k-rat tendons were also significantly larger than those of the rat, nearly 2 times larger (P_PL_ = 0.006, P_GAS_ = 0.002) and over 2.5 times larger (P_PL_ = 0.002, P_GAS_ = 0.002), respectively (Fig. 4E,F). In summary, k-rat tendons exhibited much greater toughness and strength than rat tendons.

For the comparison of failure values between PL and GAS tendons within a species, failure load of GAS tendon was 84% and 89% larger than PL tendon for rat and k-rat, respectively (P_rat_ = 0.002, P_k-rat_ = 0.002) (Fig. 4C). Failure strain and failure stress of the GAS tendon of k-rat was 29% longer (P = 0.009) and 22% smaller (P = 0.037) than the PL, respectively, while these properties were not different between GAS and PL tendons of the rats (P_failure strain_ = 0.1, P_failure stress_ = 0.084) (Fig. 4D,E). Our results also showed that there was no significant difference in the toughness of the GAS and PL tendons within rat and k-rat groups (P_rat_ = 0.16, P_k-rat_ = 0.92) (Fig. 4F). Cumulatively, the main differences found with respect to material failure properties were between the PL and GAS tendons of the k-rat.

To analyze the pre-failure properties, the stress-strain curve was divided into a toe and elastic (*i*.*e*., linear) region at a systematically defined transition point (Fig. 2). When comparing PL and GAS tendons between the rat and k-rat groups, significant differences were only found for toe modulus of the PL tendon (P = 0.002) and toe-to-linear transition displacement (P = 0.019) and strain (P = 0.03) of the GAS. In general, for the stiffness and modulus, there were more differences between the PL and GAS tendon within a species than between the tendons of different species. Significant differences were found for elastic stiffness, elastic modulus and toe stiffness between the PL and GAS tendons within the rat and k-rat groups. Elastic stiffness of GAS tendon were 67% and 48% larger than those of PL tendon (P_rat_ = 0.006, P_k-rat_ = 0.002), while elastic modulus of GAS tendon were 35% and 37% smaller than those of the PL tendon (P_rat_ = 0.027, P_k-rat_ = 0.002), for rat and k-rat respectively (Fig. 4G,J). Toe stiffness of GAS tendon was larger than PL (P_rat_ = 0.004, P_k-rat_ = 0.006) (Fig. 4H) in both groups, while the toe modulus was only significantly larger for PL tendon of k-rat group (P = 0.002) (Fig. 4K). Both toe-to-linear transition displacement (P = 0.002) and strain (P = 0.002) of the GAS tendon were significantly longer than those of the PL only in the rat, while there was no significant difference for these properties between the GAS tendons of the two different species (P_trans disp_ = 0.32, P_trans strain_ = 0.32) (Fig. 4I,L).

## Discussion

The study objective was to evaluate and compare the mechanical properties of k-rat tendons to those of rats, motivated by the remarkable ability of k-rats to produce extreme jump heights. In addition to the direct comparison between the structural and material properties of k-rat tendons and those of rats, we used a comparative approach to understand the results within the context of how tendon properties may be adapted to achieve high performance motor function.

### Study limitations

We were limited to 10 samples because the k-rats samples used in this study were from wild caught animals. Thus, we performed a non-normality test and used the Wilcoxon test, which is appropriate for small sample sizes with a non-normal distribution. Further, we performed a power analysis on all comparisons (Fig. 4). The calculated powers ranged from 0.05 to 1 with the mean of 0.82 ± 0.04. However, the power of 73% of the comparisons were equal to or greater than 0.8. The power of the most distinguishing properties between rat and k-rat, namely, the failure properties and elastic modulus, was at worse 0.97. Therefore, the power analysis indicated that the statistics reported were sufficient to support the conclusions of the study.

Another limitation was we could not account for the effect of sex due to the small sample size. Sex differences have been indicated in the mechanical properties of white rat Achilles tendons^11^. In contrast, several other studies, mostly on humans^12,13^ and mice^14^, have suggested no significant differences between sexes for tendon mechanical properties. We would need more samples to make a reliable conclusion about the effect of sex on tendon mechanical properties in this study.

Measuring the CSA of these small, soft tissues is a challenge for studies that aim to determine material properties. CSA measurements may be prone to error from issues such as, assumptions about ellipsoidal geometry, non-uniform CSA along the length, gripping or dissection artifacts, and measurement technique. In the current study, one researcher performed the CSA measurements and calculations exactly in the same way for all samples to make sure they were consistent. CSAs measured here for the rat tendons were consistent with those reported in other studies after accounting for the relation between animal’s weight and the CSA of Achilles tendons^11,15–17^.

### Comparative analysis of kangaroo rat tendon properties

A challenge in using a comparative approach is that it often has to rely on previous studies to obtain enough cases to identify relationships. This consideration is especially important for tendon studies because the estimates of material properties of tendon have been shown to be sensitive to experimental parameters and methods. For example, there are multiple methodologies to estimate the CSA and initial length of the sample, and these two quantities substantially affect the estimated material properties^18^. More specifically, when comparing the results of different studies of rat Achilles tendon, failure stress estimates decrease with increasing CSA, failure strain increases with decreasing initial length, and elastic modulus increases by increasing the initial length^11,15–17^. In this study, CSA of each sample was measured with the same method, and to eliminate the effect of initial length, all tendons were tested from similar initial length (Fig. 4A). Thus, an advantage of our study was that we obtained the measurements and mechanical property metrics from all the tendons and species using identical equipment and methods, so that we avoided the potential for confounding experimental factors that could influence the comparison between the tendons of two species and the PL and GAS tendons.

**Figure 5:**
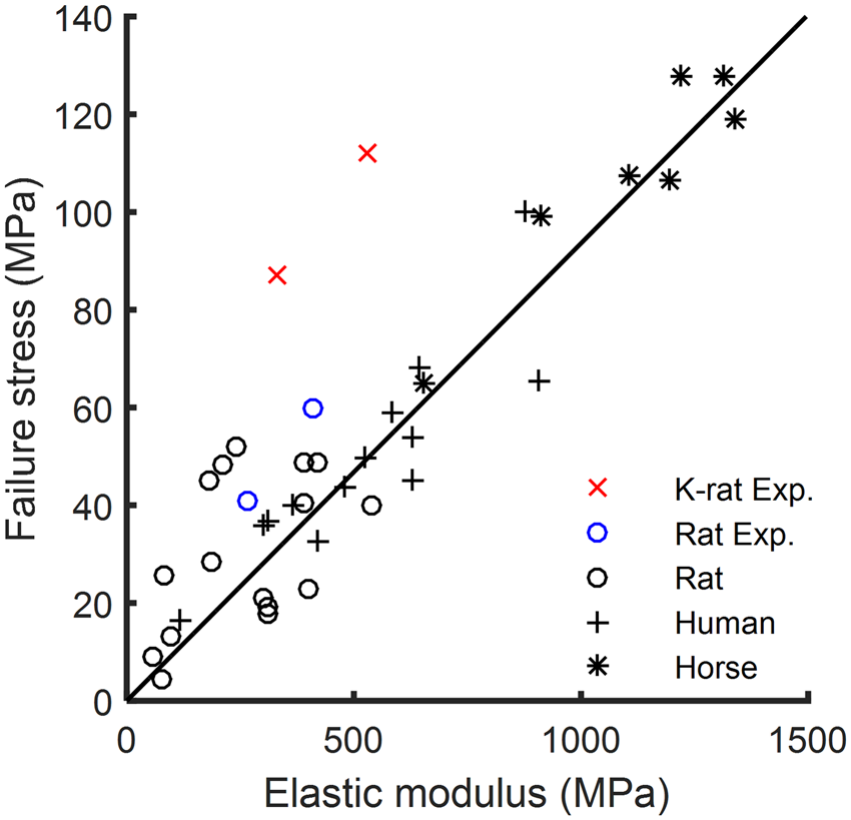
Comparative analysis. Linear relationship between failure stress and elastic modulus (line), k-rat (red x, upper cross is PL) and rat (blue o, upper circle is PL) from our experiment (K-rat Exp. and Rat Exp., respectively), and rat (black o), human (+) and horse (*) data from other studies. The plotted line is the regression calculated in the meta-analysis of healthy tendons from eleven species ranging in size from mice to cows (see Fig. 1B in reference^5^).

To our knowledge, this is the first study to report on the structural and material properties of k-rat PL and GAS tendons. A challenge when comparing these data to those of other similar species is that previous tendon studies have indicated high variability for failure stress and strain^5,19^ and elastic modulus^12,19,20^. However, as described earlier, a robust linear relationship (R^2^ = 0.785) between failure stress and elastic modulus was found through a meta-analysis of 50 studies of tendon material properties across a wide range of species^5^. To corroborate our results and to compare k-rat data with other species, we replotted the failure stress and elastic modulus of rat^5,11,15–17,21,22^, horse^5,23^ and human^5^ tendons used in the meta-analysis study and included the data obtained from our study (Fig. 5). As shown in Fig. 5, our measured failure stresses and elastic moduli of the rat PL and GAS tendons were within the range of data from previous rat tendon studies. The failure strain of rat tendons in this study (Fig. 4D) also were within the range from 9% to 25% as reported in other studies^11,15,16,21,22^. Therefore, the data obtained here for rat tendon generally agree with literature values and substantiate the methodology used.

The tendons of the k-rat in comparison to tendons of the rat had similar moduli, both in the toe and linear regions. Strikingly, the failure stress and strain of k-rat PL and GAS tendons were much larger than those of the rat, which cumulatively resulted in over 2.5 times greater toughness (Fig. 4D–F). In comparison to the values obtained in other studies (Fig. 5), the k-rat tendon strength was within the range of the strength of tendons from much larger species (*i*.*e*, horse), while k-rat tendon elastic modulus was within the range of tendon from the more comparably sized rat. These two observations clearly indicate that the k-rat tendon is an exception to the robust linear relationship of failure stress and elastic modulus found across species, ages, and other factors.

### Relevance of measured kangaroo rat tendon properties to motor function capabilities

Tendons function not only to transmit forces between muscle and skeleton, but can serve as energy storage and return elements that can increase the efficiency of the motor system^24,25^. In fact, tendons from different muscles have been classified as “energy storing” (*i*.*e*., compliant) or “positional” (*i*.*e*., stiff) depending upon whether the muscle-tendon unit is more involved with cyclic locomotion (*e*.*g*., ankle extensors) or fine positioning tasks (*e*.*g*., human digital flexors)^26^. In this study, we tested two ankle extensors, the PL and GAS, which have similar mechanical actions with regards to their moment arms, muscle fiber lengths, muscle fiber types, and muscle pennation angles^27^. Interestingly, we found that in both species tested, the elastic moduli were different between the PL and GAS tendons, which suggests that the two muscles might have different motor functions despite their mechanical similarities. For example, in addition to being an ankle extensor, in some species the PL is also a digital flexor, with the tendon extending beyond its attachment on the calcaneus to insert on the toes. However, in other species the attachment of the PL to the calcaneus is rigid, limiting any ability for the PL to independently control toe movement. The extent to which the PL is active in controlling to toes is unclear for these species; however, if the PL retains some capacity for position control, having a higher elastic modulus would be beneficial.

The ratio of muscle fiber length to tendon length is an important factor that influences elastic energy storage and recovery^28,29^. Moreover, the ability to store and return energy also depends upon both the CSA and material properties (namely, elastic modulus) of the tendon. In other words, three factors are important for the ability to store and return energy: muscle to tendon length ratio, CSA, and elastic modulus. Previous studies have indicated that ankle extensors of many large terrestrial species have evolved muscles with short muscle fibers and long tendons, ideally suited for the storage and return of elastic energy in each stride cycle^10,27,30^. We found that the k-rat muscle-tendon unit is comparable to larger species in this regard (Fig. 1). In contrast, in comparison to larger bipedal hoppers such as red kangaroos and wallabies, the Achilles tendon of the k-rats in proportion to their size are thicker^8,31^, which is not desirable for energy storage and recovery during hopping^32,33^. We found the elastic modulus of k-rat tendon not to be different (P_PL_ = 0.28, P_GAS_ = 0.084) than rat tendon (Fig. 4J), and similar to other small sized species (Fig. 5). All three factors (*i*.*e*., length, CSA, and linear modulus) contribute to the structural property of stiffness, which we found not to be different between k-rat and rat tendons (Fig. 4G). Thus, our results indicate that the k-rat tendon is no better suited for energy storage and return than the rat tendon, but this will need to be evaluated in a future study. Functionally, it appears that the k-rat tendon does support some energy storage, but also supports the ability to directly transmit work from the muscle to the skeleton and enable jumping that is important for predator escape and social behaviors^34^.

Besides transmitting forces and/or storing energy, the tendons must be able to withstand the stresses and strains encountered during motor function without failing. Moreover, it has been suggested that most of the biological safety factors (ratio of maximal failure value to maximal value under physiological conditions) fall in the range of 3–5^35^. Stresses in the Achilles tendon of k-rats have been measured *in vivo* during hopping and jumping and found to be as large as 36 MPa for the highest jumps^31^. It should be noted that even though the animal masses were similar in both studies (mean 107 versus 101 g), the average Achilles tendon CSA (PL and GAS together) reported in the previous k-rat study was approximately 50% larger than the mean value found in the current study, potentially due to methodological differences. Thus, 36 MPa could be a conservative estimate of the maximal tendon stress. Using this conservative estimate, the approximate safety factor in the GAS tendon (using the mean failure stress measured here) is 2.5. Given that jump heights in the laboratory could be substantially less than those in the wild and the high frequency that k-rats produce high jumps in the wild^34^, this estimate of the safety factor seems reasonable. Lastly, the functional implication of longer failure strain of k-rat tendon, which also contributes to greater toughness, is unclear. High extensibility of k-rat tendons might be essential for allowing the hindlimb muscles to generate optimal power during jumps^36^, but this hypothesis needs more investigation.

### Kangaroo rat tendon as a guide for better engineered tendons and biomaterials

Tendon tissue engineering and biomaterial development are motivated by the increasing incidence of tendon ruptures^37^ and poor regenerative healing that results in long-term functional deficiencies^38^. A challenge for tendon repairs is the lack of mechanically functional tendon replacements, possibly due to a limited understanding of the contributors to tendon’s mechanical properties. Our results show that the elastic modulus and strength of k-rat tendons are not linked in the same manner as the other species typically studied (*e*.*g*., mice, rats, and humans) (Fig. 5). K-rat PL and GAS tendons appear to be unique and may have evolved to be stronger and tougher compared to a wide range of tendons. Hence, k-rat tendon is an important new model that warrants further study for understanding the mechanisms that contribute to their unique mechanical properties. Contributors to the functional properties of tendon in other species have been explored and include collagen fibril continuity and structure^39^, interfibrillar sliding^40^, the nanoscale structure of collagen fibrils^41^, interfascicular matrix^42^, and the composition of the extracellular matrix ranging from elastin^43^ to collagen cross-linking^44^. Future studies exploring these potential contributors to k-rat tendon properties will be needed for understanding how these tendons achieve their extraordinary strength and toughness. Using k-rat tendon as a model and identifying these mechanisms could open new pathways for tuning structural organization and material composition to regulate the strength and toughness of tissue scaffolds and biomaterials to develop functional engineered tendons.

## Materials and Methods

### Animals

All procedures were approved by and performed in accordance to the Institutional Animal Care and Use Committee (IACUC) at Washington State University. Experiments were performed on tendons of the GAS and PL muscles of 10 adult rats (4 males, 6 females, mass = 318 ± 29.2 g, ages are between 11 to 13 months) and 10 wild-caught adult k-rats (*Dipodomys deserti*) (6 males, 4 females, mass = 101 ± 5.0 g, ages are unknown). Rats were obtained frozen from the Washington State University vivarium and were excess animals that had received no prior treatments. K-rat tendons were obtained after *in situ* muscle experiments and were kept frozen until mechanical testing.

### Tendon sample preparation

All sample preparations, setup of the testing, testing, and analysis of the data were conducted by the same researcher to ensure consistency of the methods. The hindlimb muscles (PL, LG and MG) of both legs with their tendons were carefully dissected from the proximal end, where aponeuroses are attached to the femur, to the distal end, where tendons are attached to the calcaneus. The length of the GAS and PL muscle-tendon units, both of which have the same origins such that their lengths are not different, was measured (L_MTU_ in Fig. 1). A bone chip was removed from the calcaneus and used in gripping of the distal end of GAS (common tendon of the LG and MG muscles) tendon. The PL muscle and its tendon was carefully dissected from GAS tendon. After measuring each tendon length, which was normalized by measured MTU length after dissection, GAS and PL muscles were separated from their tendons at the muscle-tendon junction. The two separated tendons (PL and GAS) were placed in a container of saline and were kept at −20 °C until preparation for mechanical testing^45^.

### Mechanical testing

Tendons from one of the legs were randomly assigned for mechanical testing. To reinforce the interface between grips and tendon, each end of the tendon samples was sandwiched between two small squares of sandpaper with a small amount of cyanoacrylate adhesive. Because the tendons of the k-rat were thinner than those of the rat, a longer portion of k-rat tendons were covered by the sandpaper to decrease the chance of slipping during the tensile test. Thus, although the k-rat tendons were longer *in situ*, the resulting grip-to-grip lengths of the k-rat and rat tendons were similar. Moreover, the portion of the tendon between the grips had an approximately uniform CSA (based upon visual inspection of the specimen images). The sandpaper was placed in the grips and secured to a materials testing machine (Instron, Norwood, MA (100 N load cell)). Prior to testing, front and side view images of the sample were captured for thickness and width measurements. Throughout testing, tendons were kept hydrated with saline.

Tendons were first preloaded to 0.1 N and distance between two grips were measured by calipers. PL and GAS tendons were preconditioned for 10 cycles with a strain rate of 1% per second of the preload length from 0.1 to 2 N and 0.1 to 5 N, respectively. The values of 2 N and 5 N roughly corresponded to the toe-to-linear transition point of PL and GAS tendons of both species, respectively. After preconditioning, initial length for calculation of material properties was measured as the grip-to-grip distance. Tendons were then stretched to failure with a constant strain rate equal to 1% of each sample’s initial length per second^46^. Load-extension data were collected at a sampling rate of 1000 Hz and imported into MATLAB (Mathworks, Natick MA) to calculate mechanical properties.

The cross-sectional area (CSA) of the tendon was estimated by an ellipse area formula: CSA = π(w*t/4) and *t* are the width and thickness of the tendon, respectively. Width and thickness of each sample were estimated by processing the captured images of tendons in ImageJ (National Institutes of Health, Bethesda, MD). The middle portion of all the images were processed to ensure that all measurements were made in the same location of the specimen. The parameters of failure load and extension were derived from the load-extension curve. To calculate toe and elastic stiffness and toe-to-linear transition displacement of each tendon, a bilinear curve was fitted to load-extension data from preload to 70% of failure load^46^. This was accomplished by finding the best fit for two linear regions of the load-extension curves of each sample, from preload to 70% of failure load, with the intersection of those lines defined as the toe-to-linear transition displacement.

CSA and initial length of the tendons were used to calculate material properties of failure stress, equal to failure load divided by CSA, and failure strain, equal to extension at failure load divided by initial length. Similar to stiffness parameters, toe and elastic modulus and toe-to-linear transition strain were derived from the stress-strain curve (Fig. 2). To show the differences between the muscle types and species in their tendon stress-strain curves, average stresses were calculated for all 10 trials of each tendon over a range of strains where all samples had data. The average was then smoothed by fitting a third-order polynomial. Finally, toughness at ultimate load was calculated as the area below the stress-strain curve (Fig. 2).

### Statistics

For statistical analyses, the Kolmogorov-Smirnov normality test was performed on all properties. Because the results showed non-normal distribution (P < 0.05), a two-way Friedman’s test was performed to compare differences between two factors (species and muscle type). If significant from Friedman’s test (P < 0.05), a Wilcoxon test was performed to determine significance between specific groups (P < 0.05). The power of the statistical comparisons also were calculated for all properties. All statistical analysis were performed using MATLAB (MathWorks, Natick, MA) statistical toolbox.
